# Combining the In Silico and In Vitro Assays to Identify *Strobilanthes cusia* Kuntze Bioactives against Penicillin-Resistant *Streptococcus pneumoniae*

**DOI:** 10.3390/ph16010105

**Published:** 2023-01-10

**Authors:** Xiaoyu Han, Lu Jin, Zhimin Zhao, Xinjun Xu, Shiyi Liu, Yuquan Huang, Xiaoli Liu, Yuehong Xu, Depo Yang, Wei Huang, Li Wang

**Affiliations:** 1 School of Pharmaceutical Sciences, Sun Yat-sen University, Guangzhou 510006, China; 2 Bacteriology & Antibacterial Resistance Surveillance Laboratory, Shenzhen Institute of Respiratory Diseases, Shenzhen People’s Hospital (The Second Clinical Medical College, Jinan University; The First Affiliated Hospital, Southern University of Science and Technology), Shenzhen 518001, China; 3 China Resources Sanjiu Medical & Pharmaceutical Co., Ltd, Shenzhen 518110, China; 4 Shenzhen Branch, Guangdong Laboratory of Lingnan Modern Agriculture, Key Laboratory of Synthetic Biology, Ministry of Agriculture and Rural Affairs, Agricultural Genomics Institute at Shenzhen, Chinese Academy of Agricultural Sciences, Shenzhen 518000, China; 5 Kunpeng Institute of Modern Agriculture at Foshan, Foshan 528225, China

**Keywords:** *Strobilanthes cusia*, penicillin-resistant *Streptococcus pneumoniae*, proteomics, reverse docking

## Abstract

Leaves of *Strobilanthes cusia* Kuntze (*S. cusia*) are a widely used alexipharmic Traditional Chinese Medicine (TCM) in southern China for the prevention of cold and respiratory tract infectious diseases. One of the most common bacterial pathogens in the respiratory tract is the gram-positive bacterium *Streptococcus pneumoniae*. The antibiotic resistance of colonized *S. pneumoniae* makes it a more serious threat to public health. In this study, the leaves of *S. cusia* were found to perform antibacterial effects on the penicillin-resistant *S. pneumoniae* (PRSP). Confocal assay and Transmission Electron Microscopy (TEM) monitored the diminished cell wall integrity and capsule thickness of the PRSP with treatment. The following comparative proteomics analysis revealed that the glycometabolism-related pathways were enriched for the differentially expressed proteins between the samples with treatment and the control. To further delve into the specific single effective compound, the bio-active contents of leaves of *S. cusia* were analyzed by UPLC-UV-ESI-Q-TOF/MS, and 23 compounds were isolated for anti-PRSP screening. Among them, Tryptanthrin demonstrated the most promising effect, and it possibly inhibited the N-glycan degradation proteins, as suggested by reverse docking analysis in silico and further experimental verification by the surface plasmon resonance assay (SPR). Our study provided a research foundation for applications of the leaves of *S. cusia* as a TCM, and supplied a bio-active compound Tryptanthrin as a candidate drug skeleton for infectious diseases caused by the PRSP.

## 1. Introduction

A leading cause of lower respiratory tract infection (LRTI) is the gram-positive bacterium *Streptococcus pneumoniae* [1], which is a common colonizer of the human nasopharynx [2]. According to a systematic analysis, 1.2 in 2.4 million deaths among global LRTI patients were attributed to *S. pneumoniae* in 2016 [3]. Especially among children under 5 years old, *S. pneumoniae* was recognized as the most fatal pathogen in 33 bacterial genera or species based on globally collected data in 2019 [4]. Both the morbidity and mortality of *S. pneumoniae* also made it a major risk of preceding viral infections, especially with influenza A virus [5]. In 2017, the World Health Organization published a priority pathogen list for new antimicrobial drugs, and penicillin-resistant *Streptococcus pneumoniae* (PRSP) was ranked 10th on the threat list. The ongoing COVID-19 pandemic has caused more than 6.2 million deaths worldwide (World Health Organization, 2022), with numbers still increasing. Its disease symptoms with a massive inflammatory immune response in the lower respiratory tract were similar to influenza, and prophylactic use of antibiotics had increased in those infected patients, which might lead to higher risk of antibiotic resistance of the colonized *S. pneumoniae* [6]. Therefore, a predisposition for PRSP infections is considered much more likely under the current global situation.

Traditional Chinese Medicine (TCM) has a very long history of clinical application. Tissues of *Strobilanthes cusia* Kuntze (*S. cusia*) have been widely used in TCM. Its root and rhizome, known as the “Southern Banlangen”, are widely used for the treatment of many epidemic diseases, such as SARS and H1N1 [7,8]. Its stem and leaf are the materials of Indigo Naturalis, known as the “Qing Dai”, one of the most commonly used alexipharmic prescriptions for respiratory tract infection in southern China [8,9]. The leaves of *S. cusia* mainly contain alkaloids, terpenoids, steroids, phenylethanol, and some other compounds, which have various pharmacological activities, such as antibacterial, antiviral, anti-inflammatory, etc. [8,10]. Previous studies focusing on the antimicrobial and antifungal activity of *S. cusia* found that the extract of Indigo Naturalis inhibited *Staphylococcus aureus*, *Staphylococcus epidermis*, *Aspergillus fumigates*, *Candida albicans*, and Methicillin-resistant *S. aureus* (MRSA) [11]; extract of roots of *S. cusia* inhibited *Escherichia coli*, *Staphylococcus aureus*, and *Bacillus subtilis* [12]; furthermore, the bio-active compounds of *S. cusia* such as Tryptanthrin were proven to inhibit *Trichophyton rubrum*, *Bacillus subtilis*, *Bacillus polymyxa* [13], *Helicobacter pylori*, and *Toxoplasma gondii*; another effective compound, N′-β-D-Glucopyranosylindirubin, inhibited *Staphylococcus aureus* [8,14]. However, the effects of *S. cusia* against *S. pneumoniae* have not been investigated yet, let alone the PRSP, and the mechanism of antibacterial effect of *S. cusia* remains unclear. Although Tryptanthrin was recognized as one of the main active compounds of *S. cusia*, its potential target against bacteria still awaits further exploration.

Since TCM is considered as a mixture of multiple chemical compounds and often targets multiple proteins and pathways, its mechanism is difficult to be explained by the modern medicine system. With the development of omics research, like genomics, proteomics and metabolomics, ways of combining bioinformatics analysis and medicinal experiments have become more and more popular and feasible to explore the obscure mechanism of TCM [15]. For example, the antimicrobial activity of epigallocatechin gallate from green tea against *Streptococcus suis* was analyzed by the synthesis of proteomics and TEM observation to infer a possible mechanism about cell wall and membrane damage [16]. In other research, the antibacterial mechanism of anthocyanins (isolated from *Aronia melanocarpa*) against *Escherichia coli* was also investigated by the combination of proteomics and TEM observation to discover that oxidative damage to the cells occurred after treatment [17]. A few studies employed in silico target fishing combined with experimental verification by the surface plasmon resonance assay (SPR) in order to find potential targets of a known antibacterial chemical or to screen out potential novel drugs for a known antibacterial target [18].

In our study, the ethanol extract of leaves from *S. cusia* (ES) were found to perform antibacterial effect on the penicillin-resistant *S. pneumoniae* (PRSP). The antibacterial mechanism of ES against PRSP were analyzed by the comparative proteomics combined with TEM and Confocal assay. To figure out the main effective chemical compound of ES, we screened anti-PRSP effect for both of the main chemical contents analyzed by UPLC-UV-ESI-Q-TOF/MS and the isolated single compounds, then selected Tryptanthrin for further exploration. Given that reverse docking is one of the in silico strategies employed in target fishing as a receptor-based high throughput screening [19], we evaluated the possible binding mode of Tryptanthrin within a database of known structural proteins of *S. pneumoniae* by reverse docking, and screened out proteins with strong binding affinity as potential targets of Tryptanthrin, which was further assessed by the SPR assay. In short, we unveiled a possible mechanism of how the ES inhibited PRSP’s growth and provided an integrated method to explore its bio-active chemical and its potentially targeted proteins of the bacteria.

## 2. Results

### 2.1. Antibacterial Activity of ES In Vitro

#### 2.1.1. Antibacterial Effect against Standard Strains

The minimal inhibitory concentration (MIC) value of ES against *S. aureus* ATCC 29213 and *S. pneumoniae* ATCC 49619 was 100 μg/mL and 200 μg/mL, respectively (Table 1). Other strains were non-susceptible (MIC > 800 μg/mL) to the treatment.

#### 2.1.2. Antibacterial Activity against Multidrug-Resistant Clinical Isolates

To further detect antibacterial effects of ES on drug-resistant strains, we tested clinical isolates PRSP and MRSA. Surprisingly, the PRSP were relatively susceptible to the treatment as the MICs were 200 μg/mL for strain F3368, F3401, F3755, and F3983. The MRSA isolates were non-susceptible, as the MICs > 800 μg/mL to the treatment for strain 48900, 49008, 48973, 49025, 48966, and 48706 (Table 2).

### 2.2. ES Inhibits the Growth of PRSP F3983

#### 2.2.1. The Growth Curves of F3983 Treated by ES

**Figure 1:**
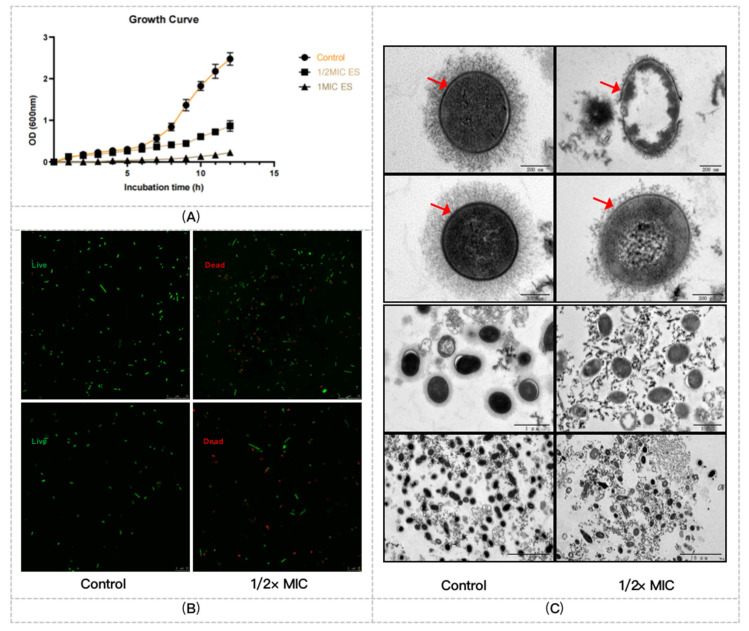
Antibacterial effects of ES on the PRSP F3983. (**A**) Growth curve of PRSP F3983 treated by ES at 1/2× MIC and 1× MIC. (**B**) Representative confocal laser scanning microscopy (CLSM) images of F3983 treated with ES 8 h at 1/2× MIC value comparing with control. The viable bacterial cells were stained green by SYTO9, and the dead cells were stained red by PI. Scales on the images are 25 µm (top two images) and 10 µm (bottom two images) as marked on. (**C**) Transmission electron microscopy images of F3983 incubated with the ES at 1/2× MIC value for 8 h compared with control. Red arrowheads point to bacteria with reduced capsule polysaccharides and damaged cell walls. Scales on the images are 200 nm (top four images), 1 µm (medium lower two images), and 5 µm (bottom two images) as marked on.

As shown in Figure 1A, 12 h incubation with ES at 1/2× MIC and 1× MIC, growth of PRSP F3983 was significantly inhibited compared with the control group.

#### 2.2.2. ES Effects on Membrane Integrity, Cell Wall, and Capsule Formation of F3983

We found that the ES disrupted the integrity of the cell wall and membrane of PRSP at 1/2× MIC, as the red color fluorescent staining cells (Figure 1B). We also observed changes at a qualitative level, in the presence, morphology, and/or thickness of pneumococcal capsule of PRSP F3983 using transmission electron microscopy. Treated PRSP F3983 cells clearly possessed a smaller amount of capsule and damaged cell wall when compared with untreated cells (Figure 1C).

### 2.3. Proteomics Analysis of PRSP F3983 Treated by ES

#### 2.3.1. Differentially Expressed Proteins (DEPs)

**Figure 2:**
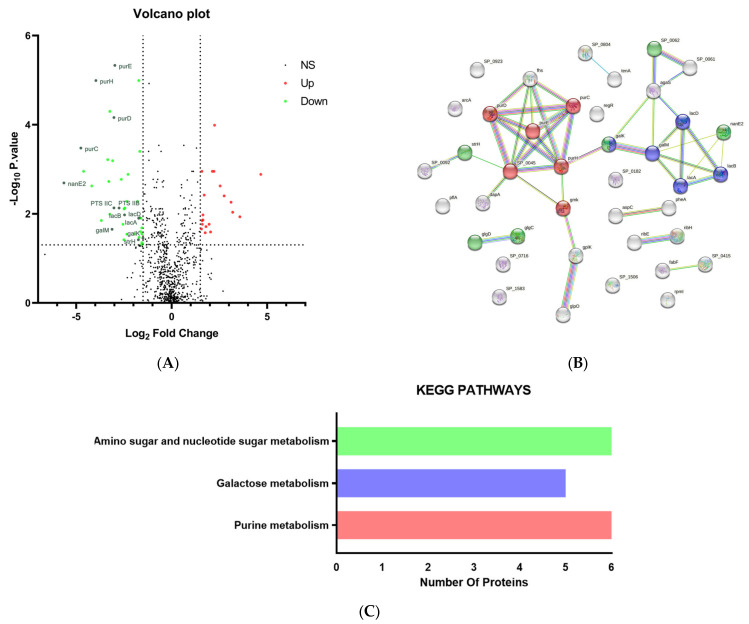
Proteomics analysis of PRSP F3983 treated by ES compared with control. (**A**) Volcano plot of the differently expressed proteins (DEPs) between control and treatment samples. (**B**) Network map of interactions among the down-regulated DEPs. The strength of interaction is represented by the line thickness, and thicker lines represent interactions with higher confidence. Each network node represents a protein; each edge denotes protein–protein associations and disconnected nodes are hidden in the network. The green, blue, and red circle colors represent proteins enriched in amino sugar and nucleotide sugar metabolism, galactose metabolism, and purine metabolism, respectively. (**C**) The enriched KEGG pathway of the down-regulated DEPs. The number of proteins included in the corresponding pathway is denoted with horizontal bars.

Sixty proteins exhibited significantly different expression (*p* < 0.05), containing 20 up-regulated proteins and 40 down-regulated proteins (Figure 2A). Detailed information of DEPs is listed in Table S1. The up-regulated proteins were not enriched on any KEGG pathway. Some of the down-regulated proteins were enriched on glycometabolism or purine metabolism-related pathways, which were partially marked on the volcano plot as the strH, PTS IIB (SP_0061), PTS IIC (SP_0062), galK, galM, LacA, LacB, LacD, nanE2, PurC, PurD, PurE, PurH. The 20 up-regulated proteins belonged to a large variety of protein families, and possessed diverse functions, such as drug resistance (ABC transport), bacterial metabolism (Methionine aminopeptidase, L-lactate dehydrogenase, Glutamate dehydrogenase), and DNA reparation (DNA topoisomerase 1). Unlike the 40 down-regulated proteins, the following enrichment analysis did not highlight any related signaling pathway (logP > 0.05). Therefore, we focused on the down-regulated proteins for further analysis.

#### 2.3.2. Protein–Protein Interactions (PPI) Analysis for Down-Regulated DEPs

The PPI network of down-regulated treatment/control group contained in total 40 nodes and 44 edges, and the average node degree was 2.2 (Figure 2B). The expected number of edges was 16, much lower than the actual quantity of 44, which means that there are more interactions among proteins than a random set of proteins with similar size. Thus, we obtained a PPI enrichment *p*-value of 9.33 × 10^−9^.

#### 2.3.3. Kyoto Encyclopedia of Genes and Genomes (KEGG) Pathway Analysis of the Down-Regulated DEPs

The enrichment and signaling pathway analysis revealed the down-regulated DEPs belonged to three metabolic pathways (Figure 2C), including purine metabolism, galactose metabolism, amino sugar, and nucleotide sugar metabolism. These pathways were related to carbohydrate metabolism and amino acid metabolism. The proteomics results, together with the evidence shown by TEM Topography figures, suggested that ES mainly affected the cell wall, capsule, and glycan biosynthesis in the PRSP F3983.

### 2.4. Chemical Contents of S. cusia Leaves and Antibacterial Effects on the PRSP F3983

#### 2.4.1. Chemical Contents of *S. cusia* Leaves

**Figure 3:**
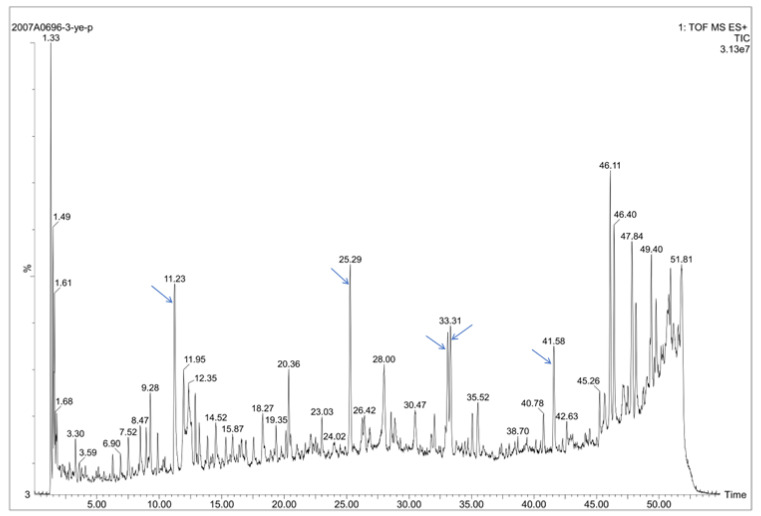
UPLC-UV-ESI-Q-TOF of *S. cusia* leaves. Blue arrows from left to right point out five main peaks at 11.23 min, 25.29 min, 33.10 min, 33.33 min, and 41.58 min, which represent CQA (includes 3-CQA, 4-CQA, 5-CQA), Hispiduloside, Tryptanthrin, Hispidulin, and Indirubin, respectively.

The chemical compounds of *S. cusia* leaves were extracted by 95% methanol and analyzed by UPLC-UV-ESI-Q-TOF/MS. Overall, 74 compounds are defined in Table S2. Five main peaks at 11.23 min, 25.29 min, 33.10 min, 33.33 min, and 41.58 min were analyzed by MS/MS fragments according to previous studies (Figure 3, Table S2), and defined as the caffeoyl quinic acid (CQA) [20,21], Hispiduloside [22], Tryptanthrin [23,24], Hispidulin [22] and Indirubin [25,26], respectively. Among them, the Tryptanthrin (peak at 33.1 min) showed the highest effective inhibition on the PRSP F3983 with MIC 25 µg/mL (Table 3). It was also reported as a bio-active chemical from plants with antiviral and antibacterial effects [27,28].

#### 2.4.2. MIC Tests for the Isolated 23 Chemical Compounds on the PRSP F3983

Overall, 23 compounds were isolated and identified by the NMR from the leaves of *S. cusia*. The isolated weight and structure of those compounds and their MICs on the PRSP F3983 are shown in Table S3. Among them, Tryptanthrin showed the most promising effect.

### 2.5. Anti-PRSP Effects of Tryptanthrin and Its Potential Targets

#### 2.5.1. Antibacterial Activity of Tryptanthrin

**Figure 4:**
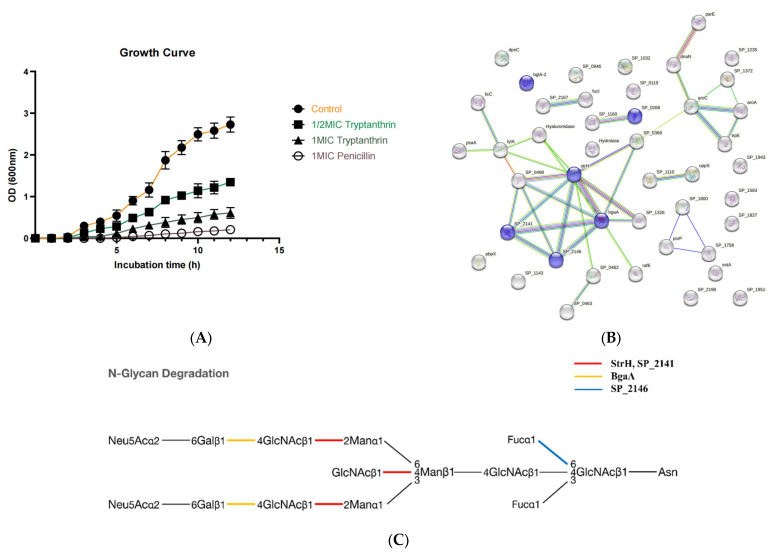
Antibacterial activity of Tryptanthrin isolated from *S. cusia* leaves. (**A**) Growth curve of F3983 treated by Tryptanthrin at 1/2× and 1× MIC. (**B**) A protein–protein interactions (PPI) network map of the selected targets. Proteins, which belong to the Glycoside hydrolase superfamily, are denoted as purple circles. (**C**) A KEGG pathway enrichment analysis for the selected targets. The red lines represent the enzymes strH and SP_2141, which release the GlcNAc; the yellow line implies the enzyme bgaA, which releases the galactose; the blue line designates the enzyme SP_2146, which releases the fucose.

The growth of PRSP F3983 was significantly inhibited by 12 h incubation with Tryptanthrin at 12.5 µg/mL and 25 µg/mL, compared with the control group. Penicillin at 2 µg/mL served as positive control (Figure 4A).

#### 2.5.2. Target Screening In Silico: Reverse Docking for Tryptanthrin with Known Structure Proteins of S. pneumoniae

To identify the potential targets of Tryptanthrin, a reverse docking-based target prediction process was employed. In our study, 623 target proteins from database of *S. pneumoniae* with their selected active pockets were analyzed (Table S4). We visualized the docking results in a probability distribution plot. Tryptanthrin had an average score of 8.000 (absolute value) with a standard deviation (SD) value of 1.534 (Table S5). We selected the top 43 candidate proteins with docking scores for Tryptanthrin. This cutoff value corresponds to the absolute average score + 0.5 SD, which referred to a protocol of reverse docking study [29]. Among them, BgaA (a beta-galactosidase), StrH (an exo-beta-D-N-acetylglucosaminidase), SP_2141 (a beta-Hexosaminidase), and SP_2146 (an alpha-fucosidase) were selected out because of their sequence similarity, which belongs to the Glycoside hydrolase superfamily (Figure 4B). Additionally, the KEGG enrichment analysis revealed that these proteins were enriched in the N-glycan degradation pathway, sharing similar substances which were GlcNAc linked polysaccharide (Figure 4C). Thus, we picked these four potential targets for further experimental verification.

### 2.6. Visualization of Tryptanthrin with Its Potential Targets by Discovery Studio

**Figure 5:**
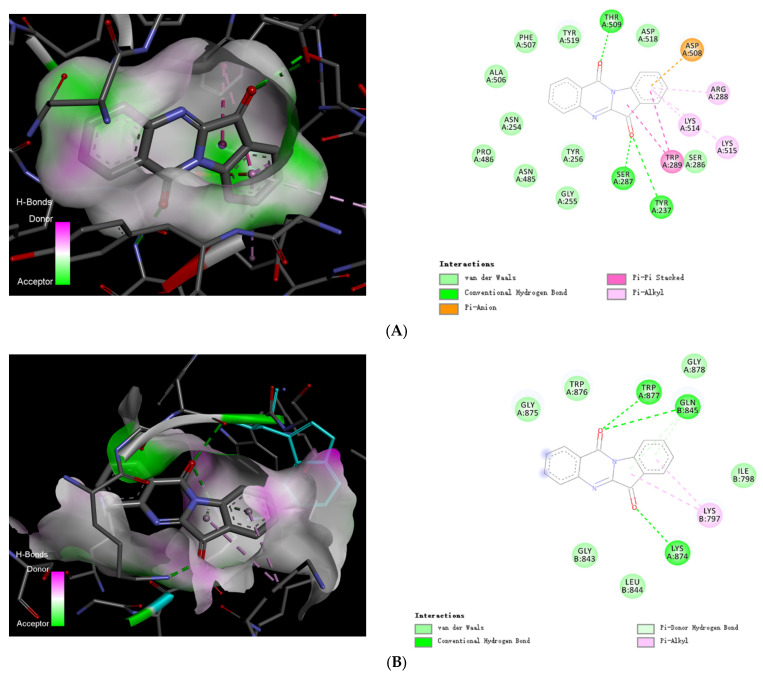

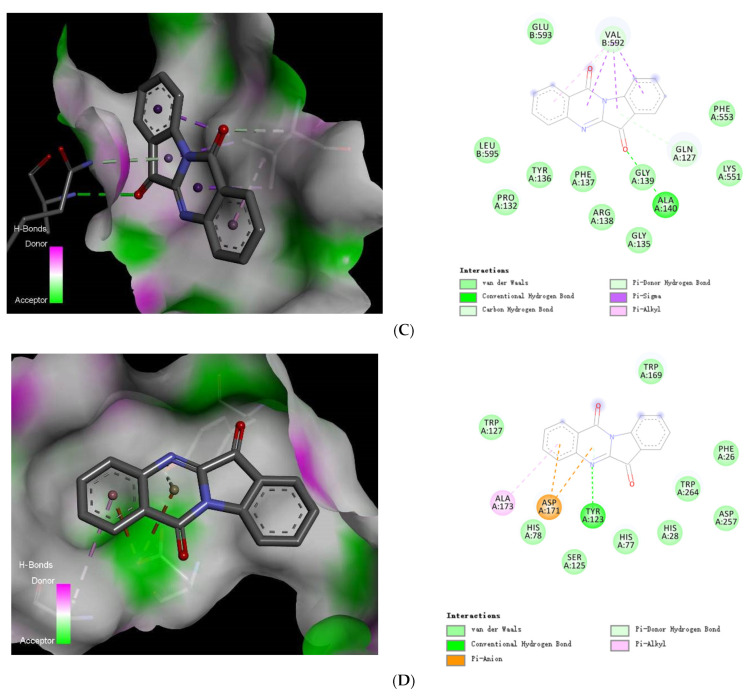
Docking conformations of Tryptanthrin with BgaA, StrH, SP_2141, and SP_2146. Diagrams representing 3D and 2D protein–ligand interactions of Tryptanthrin with the active site of BgaA (PDB code 4cu6) (**A**), StrH (PDB code 4azi) (**B**), SP_2141 (PDB code 5a6a) (**C**), and SP_2146 (PDB code 6org) (**D**).

The binding energies of Tryptanthrin to the four selected proteins BgaA, StrH, SP_2141, and SP_2146 were −9.5 kcal/mol, −9.2 kcal/mol, −8.9 kcal/mol, and −9.5 kcal/mol, respectively. Tryptanthrin intercalated into the binding pockets of each four proteins by mainly forming hydrogen bonds or aromatic stacking interactions (Figure 5).

In both cases of the BgaA and StrH, three conventional hydrogen bonds formed with ketone groups of Tryptanthrin (Indolo(2,1-b)quinazoline-6,12-dione), and hydrophobic interactions formed with indole ring. Tryptanthrin was completely trapped with BgaA by strong hydrogen bonding generated by THR509, SER287, and TYR237, with distances between 3.07 and 3.31 Å as well as Pi-Pi stack interaction with TYP289 with distances between 4.96 and 5.35 Å, and Pi-Alkyl interaction with LYS 514, LYS515, and ARG288 with distances between 4.65 and 5.34 Å, and a Pi-Charge with ASP508 with a distance of 3.20 Å (Figure 5A). It demonstrated that Tryptanthrin was connected with StrH’s active sites at TRP877, GLN845, LYS874 with strong hydrogen bonds with distances varying from 3.06 to 3.32 Å, and Pi-Alkyl interaction with LYS797 with distances between 4.76 and 5.35 Å (Figure 5B). For the protein SP_2141, Tryptanthrin showed possible interactions with its active sites, including hydrogen bonds of the ketone group on the indole ring with the GLY139, ALA140, and aromatic interactions of both the indole ring and quinazoline ring with the VAL592 (Figure 5C), indicating that the drug was possibly sandwiched between the active residues in the binding pocket. The quinazoline ring of Tryptanthrin actively formed a hydrogen bond with TRY123, as well as a Pi-Charge interaction with ASP171, and there was a Pi-Alkyl interaction with ALA173 next to it (Figure 5D).

### 2.7. Interaction of Tryptanthrin with Three Potential Targets on the N-Glycan Pathway of PRSP by Surface Plasmon Resonance (SPR) Assay

**Figure 6:**
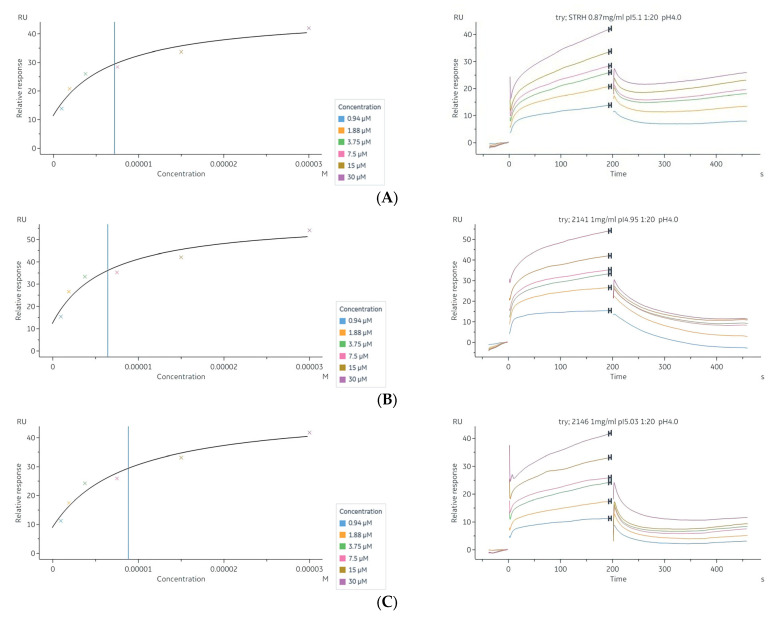
Surface plasmon resonance (SPR) analysis of the interaction between Tryptanthrin and three potential targets. (**A**) StrH KD = 7.16 × 10^−6^. (**B**) SP_2141 KD = 6.43 × 10^−6^. (**C**) SP_2146 KD = 8.82 × 10^−6^. The KD values were determined from the ratio between the kinetic rate constants (k_a_ k_d_^−1^).

To test whether the Tryptanthrin could be trapped by those glycoside hydrolase proteins detected in the reverse docking modelling, we carried out SPR assay and observed that Tryptanthrin could directly bind to the StrH, SP_2141, and SP_2146 proteins fixed on the surface of the sensor chip following a concentration-dependent manner (Figure 6, Table S6). The sequence of candidate proteins was fetched from the whole genome sequence (Bioproject number PRJNA898832) and expressed in *Escherichia coli (E. coli)* with further purification (Table S7). We determined the KDs (Steady state affinity) of Tryptanthrin binding to the StrH, SP_2141, and SP_2146, which was 7.16 μM (Figure 6A), 6.43 μM (Figure 6B), and 8.82 μM (Figure 6C), respectively. These results approved that the Tryptanthrin could bind with StrH, SP_2141, and SP_2146 automatically. For the BgaA, we failed in expressing it in *E. coli* and did not get the purified protein for SPR assay.

## 3. Discussion

According to the results of MIC selection on six common bacterial strains and ten drug-resistant clinical isolates, the leaves of *S. cusia* were proved to inhibit growth of PRSP (Figure 1A). Through the TEM photograph, the cell wall and capsule synthesis of PRSP were apparently inhibited by the treatment of 1/2× MIC ES (Figure 1C). The cell wall of *S. pneumoniae* plays a key role in cell growth and division, as well as shape maintenance. Its component peptidoglycan is cross-linked by the amidated glutamate residues in the stem peptide, and the glycan strands carry covalently attached wall teichoic acid and capsular polysaccharide [30]. Obviously, the cell wall and capsule formation of *S. pneumonia* rely on glycometabolism activities. The glycan pathways in *S. pneumoniae* have been investigated in a few studies [31,32]. A map of the cell wall and capsule biosynthesis pathways for the pneumococcal galactose, mannose, and GlcNAc catabolism was provided, and the central carbon metabolism of *S. pneumoniae* has also been studied by isotopologue profiling, which proved the biosynthesis of amino acids and a group of nucleobases or a particular nucleobase besides carbon sources were needed for capsular polysaccharide synthesis [33,34,35]. On the basis of the previous study, we deduced that ES might attenuate the expression of carbon metabolism-related proteins of PRSP.

From the results of proteomics analysis, the down-regulated proteins were enriched in three KEGG pathways, including amino sugar and nucleotide sugar metabolism, purine metabolism, and galactose metabolism (Figure 2C), which just matched the existing knowledge we found and conformed to our speculation. Combined with the morphological observation by TEM, we proposed that proteins with down-regulated expression in PRSP when treated with 1/2× MIC ES were on the glycan pathways for its cell wall and capsule synthesis. As the mapped mechanisms of the pneumococcal capsule biosynthesis shown in previous research [36], the purine metabolism work as the down-stream pathway of monosaccharide absorption, which implied that the transportation and biological utilization of sugar sources for PRSP growth [37]. As phosphorylated metabolites such as UDP-glucose and UDP-galactose are especially important as precursor molecules for capsule production [36], the down-regulated amino sugar and nucleotide sugar metabolism could be responsible for the reduction of treated PRSP’s growth and peptidoglycan synthesis. Previous research reported that the enzymes lacA, lacB, lacD, etc., were expressed on the galactose metabolism pathway [38,39], and galactose can be further metabolized in the Leloir pathway (galM, galK, etc.) [34,40]. Therefore, the down-regulated lacA, B, D, and galM, galK that we observed from the volcano plot (Figure 2A) could be explained as the reason for reduced peptidoglycan biosynthesis, which was caused by an insufficient galactose metabolism. The results of proteomics gave us a clue that some chemicals in the ES could lead to the deficiency of PRSP’s glycan metabolism, but the specific chemicals and the targeted proteins of PRSP await further exploration.

Among the identified chemical compounds of the leaves of *S. cusia* (Figure 3, Table S3), Tryptanthrin was shown to be the most effective anti-PRSP effect (MIC 25 µg/mL). Thus, it may play the predominant role of PRSP inhibition. According to previous studies of some alkaloids and flavonoids we found in the leaves of *S. cusia*, they performed antimicrobial effects on several strains. The Chlorogenic acid (3-CQA) was tested for antibacterial potential against Gram-positive (*S. aureus*, *S. pneumoniae, Bacillus subtilis*) and Gram-negative bacteria (*E. coli*, *Shigella dysenteriae*, *Salmonella Typhimurium*). The obtained MIC values ranged from 20 to 80 μg/mL [41]; 5-CQA has bactericidal effects against *P. aeruginosa*, *K. pneumoniae*, *Enterococcus faecium*, *Proteus vulgaris*, and *Candida albicans*. The obtained MIC values were in the range 1–10 mg/mL [42]. Hispidulin, purified from *Tamarix ramosissima* bark extract, showed antibacterial activity against *B. subtilis* with MIC values of 50 μg/mL and *S. aureus* with MIC values of 100 μg/mL [43]; Tryptanthrin isolated from the *Couroupita guianensis* showed promising antibacterial activity for MRSA with MIC of 62.5 μg/mL [27]. In our study, Tryptanthrin was found performing a relatively more effective antimicrobial activity against PRSP compared with other bio-active alkaloids and flavonoids identified from the leaves of *S. cusia*. To figure out its possible protein targets of PRSP and whether they are related to the deficiency of PRSP’s glycan metabolism, we performed high throughput reverse docking for the Tryptanthrin, which allowed us to discover new targets for a natural product and unveil the molecular mechanism of it [44]. The in silico calculation returned massive possibilities of potential targets of Tryptanthrin (Table S5). As we expectated, the selected candidate targets were enriched on the N-glycan degradation pathway: the BgaA, StrH, SP_2141, and SP_2146 (Figure 4B,C), as glycoside hydrolases. These super-families belong to the carbohydrate-active enzymes (CAZymes) of *S. pneumoniae*, which contain a catalytic domain to break glycosidic bonds [45].

The N-glycan glycoside hydrolases of *S. pneumoniae* are enzymes that work together to degrade complex N-linked glycans [46,47]. They have been proposed to act as nutrient acquisition and to efficiently process the complex N-glycans of its host [48,49,50]. The β-galactosidase, BgaA, is the largest cell surface attached proteins of *S. pneumoniae* for releasing galactose from complex carbohydrates [51]. StrH is an exo-β-d-N-acetylglucosaminidase able to release terminal N-acetylglucosamine (GlcNAc) residues [52]. The SP_2141 displays both N-acetylgalactosaminidase and N-acetylglucosaminidase activity [53]. The SP_2146 is cell wall-associated and responsible for the fucosylated glycan degradation to release free fucose, which is considered a previous step to release some other monosaccharides that could be imported into *S. pneumoniae* as nutrition [54].

**Figure 7:**
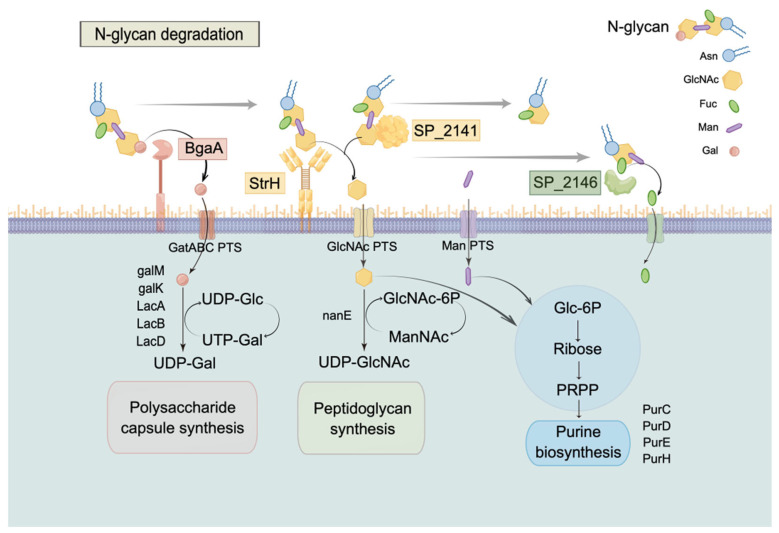
Schematic diagram of the inhibition of glycan biosynthesis by Tryptanthrin. Figure was illustrated by Figdraw (https://www.figdraw.com/, accessed on 5 November 2022). Above the membrane line, the bgaA, strH, SP_2141, and SP_2146 exhibited their function of hydrolyzation on the N-glycan degradation pathway. Below the membrane line, the monosaccharides were transported through PTS proteins into intracellular cells for peptidoglycan biosynthesis. The down-stream proteins related to carbon metabolism and cell wall or polysaccharide capsule biosynthesis were named as galK, galM, LacA, LacB, LacD, nanE, PurC, PurD, PurE, and PurH, which were also marked as down-regulated proteins in the volcano plot of proteomics analysis.

As introduced above, all of the BgaA, StrH, SP_2141, and SP_2146 of PRSP have been shown to deglycosylate N-linked glycoproteins and contribute to the ability of *S. pneumoniae* to grow on N-glycans, thus providing evidence for their role in nutrient acquisition [46,55,56]. Consistent with its ability to degrade glycans, the *S. pneumoniae* genome also encodes some carbohydrate transport systems [56], like the phosphoenolpyruvate, sugar phosphotransferase system (PTS) class. That also explained the down-regulated expression of PTS IIB and PTS IIC with ES treatment (Figure 2A), which was possibly attributed to the blocked up-stream proteins (N-glycan glycoside hydrolases) of PRSP. Combining the evidence of potential targets detected by docking Tryptanthrin, the mechanism of glycan synthesis deficiency of PRSP could be hypothesized as follows (Figure 7): Tryptanthrin inhibited the cell wall associated proteins BgaA and StrH, as well as SP_2141 and SP_2146 of PRSP, which caused the N-glycan-like carbohydrates in the broth to not be degraded and transported in to PRSP for monosaccharides assumption, with consequences of lacking raw materials for peptidoglycan biosynthesis and cell growth.

In conclusion, we found that the ethanol extract of *S. cusia* leaves, as well as its active compound Tryptanthrin, could inhibit PRSP’s growth. Tryptanthrin possibly targeted N-glycan degradation-related proteins and led to deficiency of cell wall and capsule biosynthesis of PRSP. We assumed that the down-regulated purine metabolism, galactose metabolism, phosphotransferase system (PTS), amino sugar, and nucleotide sugar metabolism were possibly affected by the inhibition of up-stream targets we found from reverse docking of Tryptanthrin, including the N-glycan degradation proteins BgaA (4cu6), StrH (4azi), SP_2141 (5a6a), and SP_2146 (6org). Given that during both colonization and invasive disease, PRSP ferments host-derived carbohydrates as its primary means of generating energy [2], our findings also indicated that ES inhibited part of nutrient acquisition function of PRSP by blocking its N-glycan degradation enzymes, which therefore diminished available monosaccharide for its own growth, especially for the cell wall and capsule synthesis. Our study not only provided the framework for future exploration to assess the mechanism of *S. cusia* leaves on clinical prophylaxis of PRSP caused infection, but also set up an example to delve into the pharmacological mechanism of TCM from both the crude extract and the single compound.

## 4. Materials and Methods

### 4.1. Preparation of Ethanol Extract of S. cusia Leaves

#### 4.1.1. Samples Collection

Total *S. cusia* samples were farmed in PingYuan County, MeiZhou City, GuangDong Province, China, and were identified by one of the authors, DY. A voucher specimen (accession number: 201904-SC-1) was deposited at the School of Pharmaceutical Sciences, Sun Yat-sen University. Their leaves were collected, air-dried, and powdered.

#### 4.1.2. Sample Preparation

Air-dried sample powder (200 g, passed through an 850 μm mesh sieve) was soaked overnight and then ultrasonically extracted with 1 L 95% ethanol for 2 h. After filtration, the extraction was concentrated by rotary evaporation.

### 4.2. UPLC-UV-ESI-Q-TOF/MS Analysis of S. cusia Leaves

Samples of *S. cusia* leaves were ultrasonically extracted with 95% methanol and filtered through a 0.22 µm membrane filters prior to UPLC analysis. Methanol and formic acid (HPLC grade) were purchased from Thermo Fisher Scientific (Waltham, MA, USA). Ultrapure water was purified using a Milli-Q system (Millipore; Boston, MA, USA). Other reagents were of analytical grade. The UPLC-ESI-Q-TOF/MS system comprises a Waters ACQUITY UPLCTM I-Class (Waters Corp., Milford, MA, USA) and a Xevo G2-XS Q-TOF-MS. A Waters ACQUITY UPLC HSS TS column (150 mm × 3.0 mm, 1.8 µm) from Waters was used. The mobile phase consisted of (A) MeOH; (B) Water containing 0.1% formic acid. Gradient elution for UPLC-Q-TOF/MS profiling was performed as follows: (0 min, 10% A; 0~35 min, 10~65% A; 35~50 min, 65~90% A; 50~51 min, 90~10% A; 51~55 min, 10% A) with a flow rate at 0.4 mL/min on 30 °C. 2 µL sample solution was injected. The detection wavelength was set at 254 nm. UPLC-Q-TOF/MS analysis was performed to identify the marker and unknown compounds in positive mode.

### 4.3. Preparation of Isolated Compounds from S. cusia Leaves

Extraction and Isolation: The air-dried sample leaves (14.0 kg) were powdered and extracted with 95% EtOH (3 × 150 L, 7 d each). The extract (1.9 kg) was obtained by filtering the supernatant, removing the solvents, and partitioning into butanol and EtOAc to afford fractions B (198 g) and E (1607 g). Fraction E was separated by Macroporous resin D101 using gradient elution with H_2_O/MeOH (80:20 to 0:100) to afford two fractions (E.1−E.2). Fraction E.1 (225 g) was divided into 5 parts (fractions E.1.1−E.1.5) by silica gel chromatography using gradient elution with petroleum ether/EtOAc (90:10 to 0:100). The five parts of E.1 were utilized to isolate 23 biochemical compounds, which were identified by Nuclear Magnetic Resonance Spectroscopy (NMR) and listed as **1**–**23** with bolded characters. E.1.1 was purified on a Sephadex LH-20 column and HPLC with H_2_O/CH_3_CN (25:75) to afford **1** (10 mg). E.1.2 was separated using a reversed-phase column using gradient elution with H_2_O/MeOH (70:30 to 0:100) to obtain three fractions (E.1.2.1−E.1.2.3). E.1.2.1 was further purified by HPLC with H_2_O/CH_3_CN (25:75) to afford **2** (182 mg) and **3** (10 mg); E.1.2.2 was purified on a Sephadex LH-20 column to afford **4** (2 mg); then the rest from last step was further purified by HPLC with H_2_O/CH_3_CN (40:60) to afford **5** (2 mg), **6** (2 mg), **7** (16 mg). E.1.2.3 purified on a Sephadex LH-20 column and HPLC with H_2_O/MeOH (40:60) to afford **8** (1 mg) and **9** (3 mg). E.1.3 was separated using a reversed-phase column using gradient elution with H_2_O/MeOH (70:30 to 0:100) to obtain two fractions (E.1.3.1−E.1.3.2). E.1.3.1 was purified on a Sephadex LH-20 column to afford **10** (12 mg) and two fractions (E.1.3.1.1−E.1.3.1.2). E.1.3.1.1 was purified by silica gel chromatography using gradient elution with petroleum ether/EtOAc (90:10 to 0:100) and HPLC with H_2_O/CH_3_CN (40:60) to afford **11** (8 mg) and **12** (26 mg). E.1.3.1.2 was purified on a Sephadex LH-20 column and HPLC with H_2_O/MeOH (35:65) to afford **13** (26 mg) and **14** (27 mg). E.1.3.2 was purified on a Sephadex LH-20 column to afford **15** (10 mg); the rest from last step were further purified by HPLC with H_2_O/CH_3_CN (60:40) to afford **16** (7 mg), **17** (4 mg), and **18** (19 mg). E.1.4 was separated by a reversed-phase column using gradient elution with H_2_O/MeOH (70:30 to 0:100) to obtain two fractions (E.1.4.1−E.1.4.2). E.1.4.1 was purified by HPLC with H_2_O/MeOH (40:60) to afford **19** (2 mg); E.1.4.2 was purified by HPLC with H_2_O/MeOH (40:60) to afford **20** (8 mg) and **21** (3 mg). E.1.5 was purified by silica gel chromatography using gradient elution with petroleum ether/MeOH (100:10 to 0:100) and HPLC with H_2_O/CH_3_CN (40:60) to afford **22** (21 mg). E.2 was purified by silica gel chromatography using gradient elution with petroleum ether/EtOAc (100:10 to 0:100) and further purified on a Sephadex LH-20 column to afford **23** (5 mg). A scheme of the whole process was drawn in Table S8.

### 4.4. Bacterial Strains and Growth Conditions

*Acinetobacter baumannii* ATCC19606, *Pseudomonas aeruginosa* ATCC27853, *Klebsiella Pneumoniae* ATCC13883, *Enterococcus faecalis* ATCC29212, *Staphylococcus aureus* ATCC29213, Methicillin-resistant *Staphylococcus aureus* (MRSA), *Streptococcus pneumoniae* ATCC49619, PRSP were from Bacteriology & Antibacterial Resistance Surveillance Laboratory, Shenzhen Institute of Respiratory Diseases, Shenzhen People’s Hospital.

For the *A. baumannii* ATCC19606, *P. aeruginosa* ATCC27853, and *K. Pneumoniae* ATCC13883, a single bacterial colony was grown in 3 mL LB broth in the tube. For the *E. faecalis* ATCC29212, *S. aureus* ATCC29213, and MRSA, a single bacterial colony was grown in 3 mL MH broth in the tube. For the *S. pneumoniae* ATCC49619 and PRSP, a single bacterial colony was grown in 3 mL CAMHB broth (Mueller Hinton II Broth, cation-adjusted for calcium and magnesium ions) with 4% horse sterile and defibrinated blood in the tube. All tubes were shaken at 230 rpm at 37 °C.

### 4.5. Minimal Inhibitory Concentration (MIC) Assay

Chemical Reference Substances (CRSs) of caffeoyl quinic acid, Hispiduloside, Tryptanthrin, Hispidulin, and Indirubin were bought from Shanghai Macklin, Shanghai, China. MICs were determined by using the standard microdilution method referred to the procedures outlined by the Clinical and Laboratory Standards Institute [57]. Briefly, bacteria were grown to the logarithmic phase with a cell density of approximately 5 × 10^5^ colony-forming units (CFUs)/mL. A two-fold serial dilution of the antimicrobial agent was prepared in a 100 μL volume in a 96-well plate, then 100 μL of the bacterial suspension was added to each well. Final concentrations of ES, CRSs, and isolated compounds were designed from 800 μg/mL to 6.25 μg/mL, along with appropriate controls. All plates were incubated at 37 °C for 24 h. MIC was determined as the lowest concentration of the antibacterial agent that completely inhibited visible bacterial growth. All MICs were determined in three independent experiments, each in duplicate.

### 4.6. Whole Genome Sequencing of PRSP (Strain F3983)

#### 4.6.1. Library Preparation and Sequencing

The genomic DNA extraction from bacterial cells was obtained by A TIANamp Bacteria DNA kit (TIANGEN, Beijing, China). DNA quality was tested by an Agilent 2100 Bioanalyzer (Agilent Technologies; Palo Alto, CA, USA) and a Qubit 4.0 Fluorometer (Thermo Fisher Scientific; Waltham, MA, USA). Library preparation was performed by the NEB Next Ultra DNA Library Prep Kit for Illumina. Samples were sequenced on a MiSeq Sequencer (Illumina; San Diego, CA, USA) using MiSeq Reagent Kits v3 (2 × 300 bp; Illumina).

#### 4.6.2. Bioinformatic Procedures

Each library generated approximately 1 G of reads. Sequences were assembled and aligned using ABySS [58] and BWA [59] for short-read alignment. Alignments were sorted and indexed using Samtools [60]. The genome and annotation file (gff3) of *S. pneumoniae* (GCF_900618125.1_NCTC11032) downloaded from NCBI were used as reference. We annotated the genome sequences with Prokka [61]. Sequencing data are available on the SRA repository under the Bioproject number PRJNA898832.

### 4.7. Growth Curves of the PRSP F3983

A single bacterial colony of PRSP F3983 was grown in 3 mL CAMHB broth with 4% horse sterile and defibrinated blood in the tube. Samples were treated with two different concentrations of ES and Tryptanthrin (1/2× MIC or 1× MIC); 1× MIC Penincilin was added as positive control. The growth curves at 37 °C were recorded by measuring the absorbance at a wavelength of 600 nm every 60 min.

### 4.8. Transmission Electron Microscopy (TEM)

A pneumococcal capsule was observed using transmission electron microscopy (TEM). Cultures added 1/2× MIC concentration (100 µg/mL) of ES to incubate together served as a sample, cultures added 95% Ethanol served as a control. Briefly, overnight cultures in CAMHB broth with 4% horse sterile and defibrinated blood of PRSP F3983 were harvested by centrifugation at 5000× *g* for 10 min. The bacterial cells were washed twice and resuspended in PBS, and fixed with the fixing solution 1, 2% paraformaldehyde and 2.5% glutaraldehyde in 0.1 M cacodylate buffer (pH 7) containing 0.075% ruthenium red, for 20 min on ice. The samples were washed with cacodylate buffer containing 0.075% ruthenium red as the washing solution. The samples were fixed again with the fixing solution 1 for 3 h, washed, and then post-fixed with the fixing solution 2, 1% osmium tetroxide in cacodylate buffer containing 0.075% ruthenium red, for 1 h at room temperature [62]. After dehydration in an ascendant series of ethanol, the samples were transferred to propylene oxide and sequentially infiltrated in EMbed 812 resin (EMS; Herrliberg, Switzerland). We used the sequence propylene oxide-resin 2:1, 1:1, and 1:2 throughout 24 h. Afterwards, samples were transferred to oxide-resin for 24 h and pure resin for another 24 h. Blocks formed in resin were polymerized for 48 h at 65 °C. Finally, samples were trimmed and sectioned in an Ultracut Reicher ultramicrotome to obtain ultrathin (40–60 nm width) samples by a diamond knife. The sections were observed and photographed in a Jeol Jem 1400 Transmission Electron Microscope.

### 4.9. Bacterial Viability Assay (Confocal)

A LIVE/DEAD BacLight bacterial viability kit was used to evaluate the effects of ES on bacterial viability. 1/2× MIC concentration (100 µg/mL) of ES was added into tube to incubate with cultures as a sample, cultures added 95% Ethanol served as a control. A single bacterial colony of PRSP F3983 was grown in 6 mL CAMHB broth with 4% horse sterile and defibrinated blood in the tube for 12 h at 37 °C. The culture was harvested by centrifugation (12,000× *g* for 5 min) when the optical density at the absorbance wavelength of 600 nm (OD_600_) is 0.6–0.8. The cells of PRSP F3983 were collected and washed three times and resuspended in 0.01 M sterile saline at OD_600_ = 0.1. A final volume of 3 µL of SYTO9 (1.67 mM, 2 µL) and PI (5 mM, 1 µL) were added in each 1 mL sample and incubated at room temperature in the dark for 15 min. A confocal laser scanning microscope (Leica TCS SP8) was used to obtain fluorescent images of the stained bacteria.

### 4.10. Proteomics Analysis

#### 4.10.1. Sample Preparation

Briefly, samples of bacteria were centrifuged and collected. 100 μL Radio Immunoprecipitation Assay (RIPA) Lysis Buffer (P0013B, Beyotime Biotechnology) was added in for protein extraction. The total protein content was quantified by the BCA protein assay kit (Beyotime Biotechnology; Shanghai, China). The supernatant was prepared with ice-cold acetone (1:4, *v/v*) at 4 °C overnight, then centrifuged and washed. It was dried and resuspended in 50 μL UA buffer (8 M Urea, 0.1 M Tris/HCl, pH 8.5). 2 μL of Dithiothreitol (DTT, finally concentration 2 mM) was added, then incubated for 1.5 h at 30 °C. Later on, 13 μL of 50 mM iodoacetamide (IAA) was added and diluted to 600 μL with 50 mM NH_4_HCO_3_, which was then kept in the dark for 40 min. Finally, trypsin solution (0.25 μg/μL, Promega, Madison, WI, USA) was added into the samples at 37 °C for 12 h with a mass ratio of 1:60 (trypsin: protein) for further digestion.

#### 4.10.2. Nano-LC–MS/MS Analysis by Q-Exactive plus Orbitrap

Two groups of Peptide mixtures (sample and control, three replicates) were tested by Easy nano-LC 1200 system (Thermo Scientific, San Jose, CA, USA) using a reverse-phase column (ReproSil-Pur C18-AQ (75 μm × 3 cm, 2 μm)) with a 120-min gradient at a flow rate of 200 nL/min. MS profiling spectra were obtained on a Q-Exactive Plus mass spectrometer (Thermo Scientific, San Jose, CA, USA). The mobile phases A and B were 0.1% formic acid in LC/MS-grade water and 0.1% formic acid in 80% acetonitrile, respectively. The peptides were separated with a linear gradient 3–32% B in 95 min, 32%–100% B in 10 min, and 100% B in 15 min. Full scans were acquired over a mass range of 355–1700 at the resolution of 70,000. The MS/MS scans were acquired at a resolution of 35,000.

#### 4.10.3. Protein Identification and Data Analysis

The collected raw data (submitted on the https://www.iprox.cn/ with the project ID: PXD038886, accessed on 18 December 2022) were processed using the Proteome Discoverer software (Thermo Fisher Scientific). Proteome of *S. pneumoniae* serotype 4 (strain ATCC BAA-334/TIGR4) was downloaded from the UniProt database (16 April 2021). The proteins were selected with high FDR confidence and if their unique peptides were tested more than four. Those were considered to be differently expressed if their log2 (Fold change) > 1.5 in the treatment group compared with the control group and the adjusted *p*-value was <0.05. The DEPs were analyzed using the GO annotation and KEGG enrichment analysis. The online web platform of STRING (https://string-db.org/, accessed on 25 April 2021) was used to analysis the protein–protein interaction network of DEPs [63,64,65,66].

### 4.11. Targets Screening for Tryptanthrin by High-Throughput Reverse Docking

#### 4.11.1. Preparation of Tryptanthrin and Protein Structure

Ligands files were downloaded from ZINC database (www.zinc15.docking.org, accessed on 20 November 2021) and used the MGLTools (https://ccsb.scripps.edu/mgltools/, accessed on 3 December 2021) to transfer to pdbqt format. A 3D structure of proteins *pdb files of *S. pneumoniae* was downloaded from the Research Collaboratory for Structural Bioinformatics Protein Data Bank (rcsbPDB—https://www.rcsb.org/, accessed on 30 December 2021) and transferred to pdbqt format by Raccoon (https://autodock.scripps.edu/resources/raccoon/, accessed on 8 December 2021). The protein data set was built according to the following advanced search parameter: (1) Experimental Method: X-ray. (2) Molecule: Protein. (3) Organism: *S. pneumoniae* (TIGR4, R6, D39). (4) X-ray Resolution: 0–3.5 Å (30 November 2021).

Crystal structures of the target proteins removed water molecules, ions, and original ligands, fixed using PBDFixer (https://github.com/pandegroup/pdbfixer, accessed on 10 December 2021), and hydrogen was added using PYMOL (http://www.pymol.org, accessed on 12 December 2021). The potential binding pockets of each protein were predicted by Fpocket (http://fpocket.sourceforge.net/, accessed on 15 December 2021).

#### 4.11.2. Reverse Docking Using AutoDock Vina

The reverse docking procedure was performed as follows: (1) Using a Python script, we generated an input file for docking with 8737 pockets from 623 target proteins in our database; (2) To provide enough space for free movements of the ligand, the grid box was constructed to cover the active sites as defined using AutoDock Vina [67]. The grid points for pockets were set to 60 × 60 × 60, at a grid center of (x, y, z) regarding to a certain pocket. (3) Docking calculation was generated with the software free energy binding own scoring function. The binding affinity of the ligand was expressed in kcal/mol, and results were sorted by docking scores and arranged into a matrix form.

### 4.12. Protein Expression and Purification

#### 4.12.1. Cloning

Gene fragments encoding StrH, SP_2141, and SP_2146 were amplified by PCR from PRSP F3983 genomic DNA (PRJNA898832). Specific primers were designed by SnapGene (https://www.snapgene.com, accessed on 12 March 2022) and were used to introduce BamHI/EcoRI restriction sites (see Table S7 for primer sequences). The StrH was cloned into the pMAL-c5X vector with a maltose-binding protein (MBP) tag, while SP_2141 and SP_2146 were cloned into pET-28a (+) vector with an N-terminal 6 × His tag, respectively. Bidirectional DNA sequencing was used to verify the fidelity of each construct.

#### 4.12.2. Protein Extraction

All recombinant expression vectors were transformed into *E. coli* BL21 Star (DE3) cells (Invitrogen). Fusion proteins were produced using LB medium supplemented with Ampicillin (50 µg/mL) for the StrH or with kanamycin (50 µg/mL) for the SP_2141 and SP_2146. Briefly, bacteria with the appropriate expression plasmid were grown at 37 °C until the OD_600_ reached 0.6–0.8. Gene expression was then induced by adding isopropyl-b-D-1-thiogalacto pyranoside (IPTG) to a final concentration of 0.5 mM, and then incubated overnight at 16 °C with shaking. Cells were harvested by centrifugation and disrupted by Phenylmethanesulfonyl fluoride (PMSF) lysis buffer (1 × PBS, 1 mM PMSF). Proteins were purified by Ni2+ immobilized metal affinity chromatography, followed by size exclusion chromatography using a Sephacryl S-200 column (GE Health care, Chicago, IL, USA) and analyzed by 10% SDS-PAGE. Protein concentration was determined and stored at −80 °C in PBS.

### 4.13. Surface Plasmon Resonance (SPR) Assay to Evaluate the Protein Binding Affinity

The Biacore 8K and sensor chip CM5 were provided by the School of Pharmaceutical Sciences, Sun Yat-sen University. The determination temperature was set at 25 °C. 1 × PBS buffer was used as the running buffer to activate the chip surface. The proteins StrH, SP_2141, SP_2146 from PRSP F3983 strain dissolved in 1 × PBS were added into 10 mM sodium acetate solution with pH 4.0 for protein conjugation. The 1.05 × PBS-P+ (with 0.05% P20) buffer containing 8% DMSO was prepared as a small molecule sample running buffer. Solvent-corrected fluids of 7.5% and 8.8% DMSO were formulated. Tryptanthrin solution was diluted down 2 times at 6 concentration gradients with 8% DMSO running buffer (30 µM, 15 µM, 7.5 µM, 3.75 µM, 1.88 µM, 0.94 µM). The protein binding time was 60 s, and the analyte flow rate was 30 µL/min.

### 4.14. Molecular Docking and Visualization

The atom type and partial charge of ligand and protein was assigned with Autodock tools [68] and was exported to *pdbqt format file for molecular docking. Autodock Vina was used to predict the potential binding pose of Tryptanthrin towards the selected proteins. The potential binding pose of selected receptors was visualized by Discovery Studio (http://www.discoverystudio.net, accessed on 22 August 2022).

### 4.15. Statistical Analysis

All the quantitative analyses (MICs, growth curves, and proteomics) were performed from three independent biological replicates, and the results were summarized as the mean ± standard deviation (*n* = 3). Data of proteomics were analyzed by one-way analysis of variance (ANOVA) and Duncan multiple range tests. The difference was considered significant at *p* < 0.05.

## Figures and Tables

**Table 1:** Determination of MICs against Gram-positive and Gram-negative reference strains.

MICs (µg/mL) of ES						
Bacteria /Strain	Gram-positive bacteria			Gram-negative bacteria		
	*Staphylococcus * *aureus* ATCC29213	*Streptococcus pneumoniae* ATCC49619	*Pseudomonas* *aeruginosa* ATCC27853	*Klebsiella* *pneumoniae* ATCC13883	*Acinetobacter* *baumannii* ATCC19606	*Enterococcus * *faecalis* ATCC29212
MIC	100	200	>800	>800	>800	>800

**Table 2:** Antibacterial effect of ES on drug-resistant clinical isolates.

MICs (µg/mL) of ES										
Bacteria	Penicillin-resistant *Streptococcus pneumonia* (PRSP)				Methicillin-resistant *Staphylococcus aureus* (MRSA)					
Strain	F3368	F3401	F3755	F3983	48900	49008	48973	49025	48966	48706
MIC	200	200	200	200	>800	>800	>800	>800	>800	>800

**Table 3:** Anti-PRSP effect of seven Chemical Reference Substances (CRSs).

MICs (µg/mL) of Seven CRSs						
3-CQA	4-CQA	5-CQA	Hispiduloside	Tryptanthrin	Hispidulin	Indirubin
200	200	200	200	25	100	>800

## Data Availability

Data is contained within the article and Supplementary Materials.

## Supplementary Materials

The following supporting information can be downloaded at: https://www.mdpi.com/article/10.3390/ph16010105/s1; Table S1: Proteome_data sheet; Table S2: UPLC-UV-ESI-Q-TOF/MS analysis of *S. cusia* leaves; Table S3: NMR of 23 isolated compounds and their MICs against PRSP [69,70,71,72,73,74,75,76,77,78,79,80,81,82,83,84,85,86,87,88,89,90,91]; Table S4: Reverse-docking_active-pockets; Table S5: Reverse-docking_results_Tryptanthrin; Table S6: LMW-Kinetics-affinity_Tryptanthrin; Table S7: Primers and sequences of bgaA, StrH, SP_2141 and SP_2146 of F3983; Table S8: A scheme of Preparation of Isolated Compounds from *S. cusia* leaves

## Abbreviations

PRSP, Penicillin-resistant *Streptococcus pneumoniae*; LRTI, Lower respiratory tract infection; TCM, Traditional Chinese medicine; ES, Ethanol extract of leaves from the *Strobilanthes cusia* Kuntze; MIC, Minimal inhibitory concentration; HPLC, High Performance Liquid Chromatography; MRSA, Methicillin-resistant *Staphylococcus aureus*; OD, Optical density; TEM, Transmission electron microscopy; KEGG, Kyoto Encyclopedia of Genes and Genomes; PPI, Protein-protein interactions; DEP, Differentially expressed proteins; PTS, phosphotransferase system; NMR, Nuclear Magnetic Resonance Spectroscopy; SPR, Surface plasmon resonance; PBS, phosphate buffered saline; DTT, Dithiothreitol; IAA, Iodoacetamide; BCA, Bicinchoninic acid; DMSO, dimethyl sulfoxide; IPTG, Isopropyl-b-D-1-thiogalacto pyranoside; PMSF, Phenylmethanesulfonyl fluoride; MBP, Maltose-binding protein; RIPA, Radio Immunoprecipitation Assay; CRS, Chemical Reference Substances.
