# Supplementary material for: Combining the In Silico and In Vitro Assays to Identify Strobilanthes cusia Kuntze Bioactives against Penicillin-Resistant Streptococcus pneumoniae
Source: Pharmaceuticals (Basel). 2023 Jan 10;16(1):105. doi: 10.3390/ph16010105 (PMC9863409; doi:10.3390/ph16010105)
Supplement: Supplementary file 1 [file pharmaceuticals-16-00105-s001.zip › Table S3. NMR of 23 isolated compounds and their MICs against PRSP.pdf]

NMR of 23 compounds and their MICs against PRSP:

Tryptanthrin (1) [1], Yellow needles; ESI-MS *m/z*: 249 $[M+H]^+$; ^1H NMR (400 MHz, $CDCl_3$): δ_H 8.58 (1H, d, *J* = 8.1 Hz, H-10), 8.39 (1H, dd, *J* = 7.9, 1.1 Hz, H-1), 7.99 (1H, d, *J* = 8.1 Hz, H-4), 7.88 (1H, dd, *J* = 7.6, 0.7 Hz, H-7), 7.82 (1H, t, *J* = 7.8 Hz, H-3), 7.75 (1H, t, *J* = 7.8 Hz, H-9), 7.64 (1H, t, *J* = 7.6 Hz, H-2), 7.40 (1H, t, *J* = 7.6 Hz, H-8); ^{13}C NMR (100 MHz, $CDCl_3$): δ_C 182.8 (C-6), 158.3 (C-12), 146.8 (C-4a), 146.5 (C-10a), 144.5 (C-5a), 138.5 (C-9), 135.3 (C-3), 130.9 (C-4), 130.4 (C-2), 127.7 (C-1), 127.4 (C-8), 125.6 (C-7), 123.9 (C-12a), 122.1 (C-6a), 118.2 (C-10).

2(3H)-benzoxazolinone (2) [2], White powders; ESI-MS *m/z*: 158 $[M+Na]^+$, 134 $[M-H]^-$; ^1H NMR (400 MHz, CD_3OD): δ_H 7.07 (4H, m, H-4~7); ^{13}C NMR (100 MHz, CD_3OD): δ_C 156.4 (C-2), 143.9 (C-7a), 129.5 (C-3a), 124.2 (C-7), 122.7 (C-4), 110.3 (C-5), 110.1 (C-6).

(3*R*, 6*R*, 7*E*)-3-hydroxy-4, 7-megastigmadien-9-one (3) [3], Colorless oil; ESI-MS *m/z*: 209 $[M+H]^+$; ^1H NMR (400 MHz, $CDCl_3$): δ_H 6.54 (1H, dd, *J* = 15.8, 10.1 Hz, H-7), 6.10 (1H, d, *J* = 15.8 Hz, H-8), 5.63 (1H, s, H-4), 4.27 (1H, br s, H-3), 2.50 (1H, d, *J* = 10.1 Hz, H-6), 2.26 (3H, s, 10-CH_3), 1.84 (1H, dd, *J* = 13.5, 5.9 Hz, H-2β), 1.62 (3H, s, 13-CH_3), 1.41 (1H, dd, *J* = 13.5, 6.3 Hz, H-2α), 1.03 (3H, s, 11-CH_3), 0.89 (3H, s, 12-CH_3); ^{13}C NMR (100 MHz, $CDCl_3$): δ_C 198.3 (C-9), 147.4 (C-7), 135.6 (C-5), 133.8 (C-8), 126.0 (C-4), 65.7 (C-3), 54.5 (C-6), 44.0 (C-2), 34.1 (C-1), 29.5 (C-11), 27.4 (C-10), 24.9 (C-12).

1, 3-dihydro-2*H*-indol-2-one (4) [4], White powder; ESI-MS *m/z*: 134 $[M+H]^+$; ^1H NMR (400 MHz, $CDCl_3$): δ_H 7.20 (2H, t, *J* = 7.7 Hz, H-4, 7), 7.00 (1H, t, *J* = 7.5 Hz, H-6), 6.86 (1H, d, *J* = 7.7 Hz, H-5), 3.52 (2H, s, H-3); ^{13}C NMR (100 MHz, $CDCl_3$): δ_C 177.6 (C-2), 142.5 (C-7a), 128.1 (C-4), 125.5 (C-3a), 124.9 (C-5), 122.6 (C-6), 109.9 (C-7), 36.4 (C-3).

1*H*-pyrrole-2, 5-diacarboxylic acid (5) [5], Yellow solid; ESI-MS *m/z*: 178 $[M+Na]^+$; ^1H NMR (400 MHz, $CDCl_3$): δ_H 6.89 (2H, d, *J* = 2.6 Hz, H-3, 4); ^{13}C NMR (100 MHz, $CDCl_3$): δ_C 161.7 (2-COOH), 126.4(C-2, 5), 115.9 (C-3, 4).

bis(2-ethylhexyl) benzene-1, 2-dicarboxylate (6) [6], Yellow oil; ESI-MS *m/z*: 391 $[M+H]^+$; ^1H NMR (400 MHz, $CDCl_3$): δ_H 7.69 (2H, dd, *J* = 5.7, 3.3 Hz, H-3″, 6″), 7.51 (2H, dd, *J* = 5.7, 3.3 Hz, H-4″, 5″), 4.20 (4H, d, *J* = 5.9 Hz, 2 x O-CH_2), 1.65 (2H, m, H-2, 2′), 1.30 (16H, m, 8 x CH_2), 0.90 (6H, t, *J* = 7.5 Hz, H-8, 8′), 0.87 (6H, t, *J* = 7.0 Hz, H-6, 6′); ^{13}C NMR (100 MHz, $CDCl_3$): δ_C 168.0 (C=O), 132,7 (C-1″, 2″),131.1 (C-4″, 5″), 129.0 (C-3″, 6″), 68.4 (C-1, 1′), 38.9 (C-2, 2′), 30.6 (C-3, 3′), 29.1 (C-4, 4′), 24.0 (C-7, 7′), 23.2 (C-5, 5′), 14.3 (C-8, 8′), 11.2 (C-6, 6′).

phthalic acid isodibutyl ester (7) [7], Yellow oil; ESI-MS *m/z*: 279 $[M+H]^+$; ^{1}H NMR (400 MHz, $CDCl_3$): δ_H 7.70 (2H, m, H-3, 6), 7.51 (2H, m, H-4, 5), 4.06 (4H, d, *J* = 6.7 Hz, H-2′, 2″), 2.02 (2H, m, H-3′, 3″), 0.96 (12H, d, *J* = 6.7 Hz, H-4′, 5′, 4″, 5″); ^{13}C NMR (100 MHz, $CDCl_3$): δ_C 167.9 (C-1′, 1″), 132.6 (C-1, 2), 131.1 (C-3, 6), 129.0 (C-4, 5), 72.0 (C-2′, 2″), 27.9 (C-3′, 2″), 19.4 (C-4′, 5′, 4″, 5″).

cephalandole B (8) [8], Yellow powder; ESI-MS *m/z*: 295 $[M+H]^+$, 317 $[M+Na]^+$; ^{1}H NMR (400 MHz, $CDCl_3$): δ_H 11.72 (1H, br s, 1′-NH), 8.92 (1H, d, *J* = 8.0 Hz, H-6′), 8.55 (1H, br s, H-1), 8.40 (1H, d, *J* = 7.5 Hz, H-4), 8.06 (1H, d, *J* = 8.0 Hz, H-3′), 7.95 (1H, d, *J* = 2.6 Hz, H-2), 7.58 (1H, t, *J* = 7.9 Hz, H-5′), 7.43 (1H, d, *J* = 7.3 Hz, H-7), 7.29 (2H, m, H-5, 6), 7.07 (1H, t, *J* = 7.4 Hz, H-4′), 3.94 (3H, s, 7′-OCH_3); ^{13}C NMR (100 MHz, $CDCl_3$): δ_C 169.4 (C-7′), 163.8 (C-10), 142.7 (C-1′), 136.5 (C-8), 135.0 (C-5′), 131.1 (C-3′), 127.5 (C-2), 125.9 (C-9), 123.6 (C-6), 122.2 (C-5), 122.0 (C-4′), 121.8 (C-4), 120.7 (C-6′), 114.8 (C-2′), 113.7 (C-3), 111.6 (C-7), 52.6 (7′-OCH_3).

(-)-3-hydroxy-*β*-ionone (9) [9], Yellow oil; ESI-MS *m/z*: 209 $[M+H]^+$; ^{1}H NMR (500 MHz, CD_3OD): δ_H 7.34 (1H, d, *J* = 16.5 Hz, H-7), 6.15 (1H, d, *J* = 16.5 Hz, H-8), 3.93 (1H, m, H-3), 2.31 (3H, s, H-10), 1.80 (3H, s, H-13), 1.15 (3H, s, H-12), 1.12 (3H, s, H-11); ^{13}C NMR (125 MHz, CD_3OD): δ_C 201.4 (C-9), 144.6 (C-7), 137.0 (C-6), 134.4 (C-8), 133.4 (C-5), 65.0 (C-3), 48.8 (C-2), 43.6 (C-4), 37.9 (C-1), 30.7 (C-11), 29.0 (C-12), 27.4 (C-10), 21.9 (C-13).

4-hydroxy-5-methoxy-benzoic acid (10) [10], White powder; ESI-MS: *m/z* 167 $[M-H]^+$; ^{1}H NMR (400 MHz, CD_3OD): δ_H 7.55 (2H, m, H-2, 6), 6.83 (1H, d, *J* = 8.7 Hz, H-3), 4.90 (3H, s, 5-OCH_3); ^{13}C NMR (100 MHz, CD_3OD): δ_C 170.3 (1-COOH), 152.8 (C-4), 148.8 (C-5), 125.4 (C-2), 123.3 (C-1), 116.0 (C-3), 113.9 (C-6), 56.5 (5-OCH_3).

3*β*-hydroxy-5*α*, 6*α*-epoxy-7-megastigmen-9-one (11) [11], Colorless oil; ESI-MS *m/z*: 225 $[M+H]^+$; ^{1}H NMR (400 MHz, $CDCl_3$): δ_H 7.00 (1H, d, *J* = 15.6 Hz, H-7), 6.25 (1H, d, *J* = 15.6 Hz, H-8), 3.87 (1H, m, H-3), 2.35 (1H, dd, *J* = 14.5, 5.0 Hz, H-4*β*), 2.25 (3H, s, H-10), 1.63 (1H, m, H-4*α*), 1.59 (1H, m, H-2*α*), 1.23 (1H, m, H-2*β*), 1.16 (6H, s, H-12, 13), 0.94 (3H, s, H-11); ^{13}C NMR (100 MHz, $CDCl_3$): δ_C 197.7 (C-9), 142.7 (C-7), 132.8 (C-8), 69.7 (C-6), 67.5 (C-5), 64.1 (C-3), 46.8 (C-2), 40.7 (C-4), 35.3 (C-1), 29.5 (C-12), 28.5 (C-10), 25.1 (C-11), 20.0 (C-13).

calendin (12) [12], White powder; ESI-MS *m/z*: 197 $[M+H]^+$; ^{1}H NMR (400 MHz, $CDCl_3$): δ_H 5.65 (1H, s, H-3), 4.29 (1H, dt, *J* = 9.6, 6.4, 3.2 Hz, H-6), 2.44 (1H, dt, *J* = 13.7, 5.1, 2.6 Hz, H-7a), 2.20 (1H, d, *J* = 2.7 Hz, 6-OH), 1.96 (1H, dt, *J* = 13.7, 5.1, 2.6 Hz, H-5a), 1.75 (3H, s, 7a-CH_3), 1.71 (1H, d, *J* = 3.9 Hz, H-7b), 1.48 (1H, dd, *J* = 14.6, 3.7 Hz, H-5b), 1.43 (3H, s, 4-CH_3), 1.23 (3H, s, 4-CH_3); ^{13}C NMR (100 MHz, $CDCl_3$): δ_C 183.1 (C-3a), 172.4 (C-2), 112.9 (C-3), 87.2 (C-7a), 66.8 (C-6), 47.4 (C-5), 45.7 (C-7), 36.2 (C-4), 30.8 (C-8, 4-CH_3), 27.1 (C-10, 7a-CH_3), 26.6 (C-9, 4-CH_3).

3-(2'-hydroxyphenyl)-4(3H)-quinazolinone (13) [13], White powder; ESI-MS m/z: 239 $[M+H]^+$; 1H NMR (500 MHz, CD_3OD): δ_H 8.17 (1H, d, *J* = 7.9 Hz, H-5), 8.05 (1H, s, H-2), 7.76 (1H, t, *J* = 7.1 Hz, H-7), 7.64 (1H, d, *J* = 8.1 Hz, H-8), 7.48 (1H, t, *J* = 7.5 Hz, H-6), 7.24 (2H, m, H-4′, 6′), 6.96 (1H, d, *J* = 8.1 Hz, H-3′), 6.90 (1H, t, *J* = 7.3 Hz, H-5′); ^{13}C NMR (125 MHz, CD_3OD); δ_C 161.7 (C-4), 153.6 (C-2′), 148.7 (C-2), 148.3 (C-8a), 135.2 (C-7), 131.2 (C-4′), 129.4 (C-6′), 128.0 (C-6), 127.3 (C-8), 127.0 (C-5), 125.2 (C-1′), 122.7 (C-4a), 120.2 (C-5′), 117.1 (C-3′).

1*H*-indole-3-carbaldehyde (14) [14], White powder; ESI-MS *m/z*: 146 $[M+H]^+$; ^1H NMR (400 MHz, CD_3OD): δ_H 9.85 (1H, s, CHO), 8.14 (1H, dd, *J* = 7.0, 2.0 Hz, H-4), 8.05 (1H, s, H-2), 7.45 (1H, d, *J* = 7.4 Hz, H-7), 7.23 (2H, m, H-5, 6); ^{13}C NMR (100 MHz, CD_3OD): δ_C 187.6 (C-8), 139.8 (C-2), 139.0 (C-7a), 125.8 (C-3a), 125.1 (C-4), 123.7 (C-6), 122.5 (C-5), 120.2 (C-3), 113.2 (C-7).

hispidulin (15) [15], Yellow powder; ESI-MS *m/z*: 301 $[M+H]^+$; ^1H NMR (400 MHz, CD_3OD): δ_H 7.83 (2H, d, *J* = 7.3 Hz, H-2′, 6′), 6.91 (2H, d, *J* = 7.3 Hz, H-3′, 5′), 6.57 (1H, s, H-8), 6.54 (1H, s, H-3), 3.87 (3H, s, 6-OCH_3); ^{13}C NMR (100 MHz, CD_3OD): δ_C 184.4 (C-4), 166.5 (C-2), 162.9 (C-4′), 158.9 (C-7), 154.8 (C-5), 154.2 (C-9), 133.0 (C-6), 129.6 (C-2′, 6′), 123.4 (C-1′), 117.2 (C-3′, 5′), 105.9 (C-10), 103.5 (C-3), 95.4 (C-8), 61.1 (6-OCH_3).

2-hydroxy-(2*H*)-1, 4-benzoxazin-3(4*H*)-one (16) [16], Faint yellow amorphous; ESI-MS *m/z*: 166 $[M+H]^+$; ^1H NMR (400 MHz, CD_3OD): δ_H 6.99 (3H, m, H-6, 7, 8), 6.93 (1H, m, H-5), 5.53 (1H, s, H-2); ^{13}C NMR (100 MHz, CD_3OD): δ_C 165.4 (C-3), 142.5 (C-9), 127.6 (C-10), 125.0 (C-6), 123.9 (C-7), 118.9 (C-8), 117.0 (C-5), 92.2 (C-2).

2-hydroxy-*N*-(2-hydroxyphenyl)propionamide (17) [17], White powder; ESI-MS *m/z*: 182 $[M+H]^+$, 204 $[M+Na]^+$; ^1H NMR (400 MHz, CD_3COCD_3): δ_H 8.08 (1H, d, *J* = 7.8 Hz, H-6′), 6.93 (2H, m, H-3′, 4′), 6.82 (1H, m, H-5′), 4.31 (1H, q, *J* = 6.8 Hz, H-2), 1.43 (3H, d, *J* = 6.8 Hz, H-3), ^{13}C NMR (100 MHz, CD_3COCD_3): δ_C 174.1 (C-1), 147.7 (C-2′), 127.5 (C-1′), 125.2 (C-5′), 121.1 (C-4′), 120.6 (C-6′), 116.7 (C-3′), 69.2 (C-2), 21.3 (C-3).

bungein A (18) [18], White powder; ESI-MS *m/z*: 273 $[M-H]^-$; ^1H NMR (400 MHz, $CDCl_3$): δ_H 7.01 (4H, dd, *J* = 8.4, 2.9 Hz, H-3, 5, 3′, 5′), 6.69 (4H, dd, *J* = 8.4, 2.9 Hz, H-2, 6, 2′, 6′), 3.67 (4H, t, *J* = 7.2 Hz, H-*β*, *β*′), 2.70 (4H, t, *J* = 7.2 Hz, H-*α*, *α*′); ^{13}C NMR (100 MHz, $CDCl_3$): δ_C 156.9 (C-1,1′), 131.1 (C-4, 4′), 131.0 (C-3, 5, 3′, 5′), 116.3 (2, 6, 2′, 6′), 64.7 (C-*β*, *β*′), 39.5 (*α*, *α*′).

p-hydroxyacetophenone (19) [19], White powder; ESI-MS *m/z*: 137 $[M+H]^+$; ^1H NMR (400 MHz, CD_3COCD_3): δ_H 7.88 (2H, d, *J* = 9.0 Hz, H-2′, 6′), 6.91 (2H, d, *J* =

9.0 Hz, H-3′, 5′), 2.48 (3H, s, H-2); ^{13}C NMR (100 MHz, CD_3COCD_3): δ_C 196.3 (C-1), 162.8 (C-4′), 131.6 (C-2′, 6′), 130.6 (C-1′), 116.0 (C-3′, 5′), 26.4 (C-2).

deoxyvasicinone (20) [20], Yellow oil; ESI-MS *m/z*: 187 $[M+H]^+$; ^{1}H NMR (500 MHz, $CDCl_3$): δ_H 8.25 (1H, dd, *J* = 7.9, 0.6 Hz, H-8), 7.69 (1H, m, H-7), 7.61 (1H, d, *J* = 8.1 Hz, H-6), 7.41 (1H, t, *J* = 7.3 Hz, H-5), 4.18 (2H, t, *J* = 7.5 Hz, H-11), 3.15 (2H, t, *J* = 7.9 Hz, H-9), 2.26 (2H, m, H-10); ^{13}C NMR (125 MHz, $CDCl_3$): δ_C 161.2 (C-4), 159.7 (C-2), 149.3 (C-8a), 134.4 (C-7), 126.9 (C-6), 126.6 (C-8), 126.5 (C-5), 120.7 (C-4a), 46.7 (C-11), 32.7 (C-9), 19.7 (C-10).

Nicotinamide (21) [21], White powder; ESI-MS *m/z*: 123 $[M+H]^+$; ^{1}H NMR (500 MHz, CD_3COCD_3): δ_H 9.10 (1H, d, *J* = 1.6 Hz, H-2), 8.70 (1H, d, *J* = 3.7 Hz, H-6), 8.25 (1H, dt, *J* = 7.9, 1.8 Hz, H-4), 7.47 (1H, dd, *J* = 7.9, 4.8 Hz, H-5); ^{13}C NMR (125 MHz, CD_3COCD_3): δ_C 167.5 (C-7), 153.0 (C-2), 149.8 (C-6), 135.9 (C-4), 130.7 (C-3), 124.2 (C-5).

4(*3H*)-quinazolinone (22) [22], White needles; ESI-MS *m/z*: 147 $[M+H]^+$; ^{1}H NMR (400 MHz, C_5D_5N): δ_H 8.57 (1H, d, *J* =7.9 Hz, H-5), 8.48 (1H, s, H-2), 7.94 (1H, d, *J* = 8.1 Hz, H-7), 7.74 (1H, t, *J* = 7.6 Hz, H-8), 7.47 (1H, t, *J* = 7.5 Hz, H-6); ^{13}C NMR (100 MHz, C_5D_5N): δ_C 162.5 (C-4), 150.5 (C-8a), 146.3 (C-2), 134.9 (C-7), 128.5 (C-6), 127.4 (C-5), 127.2 (C-8), 124.6 (C-4a).

9(*Z*)-octadecenamide (23) [23], White powder; ESI-MS *m/z*: 282 $[M+H]^+$; ^{1}H NMR (400 MHz, CD_3OD): δ_H 5.34 (2H, m, H-9, 10), 2.19 (2H, t, *J* =7.2 Hz, H-2), 2.03 (4H, m, H-8, 11), 1.60 (2H, t, *J* = 7.0 Hz, H-3), 1.29 (20H, s, H-4~7, 12~17), 0.90 (3H, t, *J* = 6.8 Hz, H-18); ^{13}C NMR (100 MHz, CD_3OD): δ_C 179.7 (C-1), 131.0 (C-9, 10), 36.7 (C-2), 33.2 (C-16), 31.0 (C-7), 30.9 (C-12), 30.8 (C-6), 30.8 (C-13), 30.8 (C-14), 30.8 (C-15), 30.6 (C-5), 30.5 (C-4), 28.3 (C-8, 11), 27.1 (C-3), 23.9 (C-17), 14.6 (C-18).

Table S3. NMR of 23 isolated compounds and their MICs against PRSP.

NO.	MIC (μg/ml)	Weight (mg)	Molecular weight / molecular formula	Chemical structure and name
1	50	10	248 ($C_{15}H_8N_2O_2$)	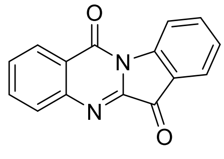 tryptanthrin
2	>800	182	135 ($C_7H_5NO_2$)	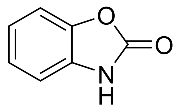 2(3H)-benzoxazolinone

3	400	10	208 ($C_{13}H_{20}O_2$)	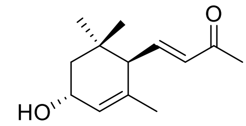 (3R,6R,7E)-3-hydroxy-4,7-megastigmadien-9-one
4	400	2	133 (C_8H_7NO)	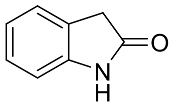 1,3-dihydro-2H-indol-2-one
5	200	2	155 ($C_6H_5NO_4$)	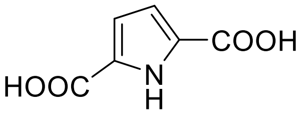 1H-pyrrole-2,5-diacarboxylic acid
6	>800	2	390 ($C_{24}H_{38}O_4$)	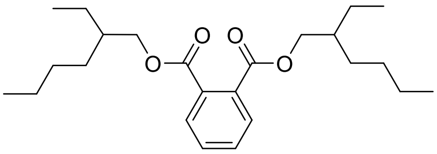 bis(2-ethylhexyl) benzene-1,2-dicarboxylate
7	>800	16	278 ($C_{16}H_{22}O_4$)	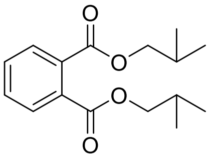 phthalic acid isodibutyl ester
8	>800	1	294 ($C_{17}H_{14}N_2O_3$)	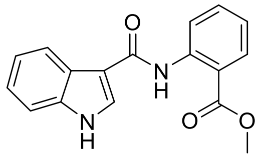 cephalandole B
9	>800	3	208 ($C_{13}H_{20}O_2$)	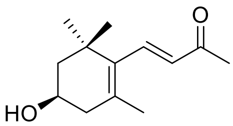 (-)-3-hydroxy-β-ionone
10	>800	12	168 ($C_8H_8O_4$)	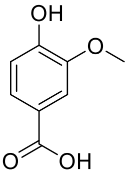 4-hydroxy-5-methoxy-benzoic acid
11	400	8	224 ($C_{13}H_{20}O_3$)	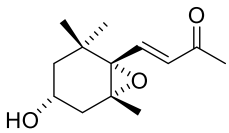 3β-hydroxy-5α,6α-epoxy-7-megastigmen-9-one

12	>800	26	196 ($C_{11}H_{16}O_3$)	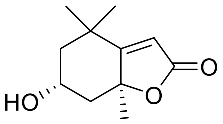 Calendin
13	400	26	238 ($C_{14}H_{10}N_2O_2$)	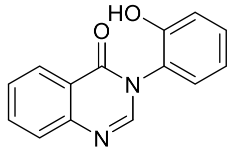 3-(2'-hydroxyphenyl)-4(3H)-quinazolinone
14	>800	27	145 (C_9H_7NO)	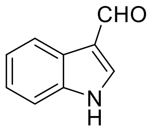 1H-indole-3-carboxadehyde
15	200	10	300 ($C_{16}H_{12}O_6$)	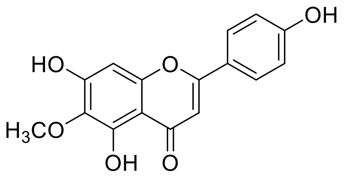 hispidulin
16	>800	7	165 ($C_8H_7NO_3$)	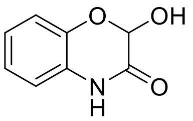 2-hydroxy-(2H)-1,4-benzoxazin-3(4H)-one
17	>800	4	181 ($C_9H_{11}NO_3$)	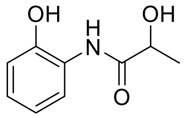 2-hydroxy-N-(2-hydroxyphenyl)propionamide
18	>800	19	274 ($C_{16}H_{18}O_4$)	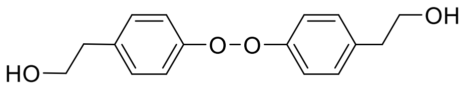 bungein A
19	200	2	136 ($C_8H_8O_2$)	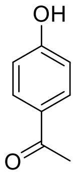 p-hydroxyacetophenone
20	>800	8	186 ($C_{11}H_{10}N_2O$)	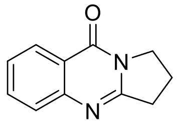 deoxyvasicinone
21	>800	3	122 ($C_6H_6N_2O$)	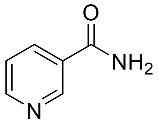 Nicotinamide

22	>800	21	146 ($C_8H_6N_2O$)	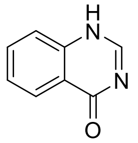<br>4(3H)-quinazolinone
23	>800	5	281 ($C_{18}H_{35}NO$)	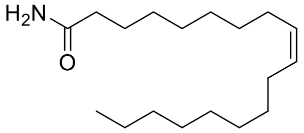<br>9(Z)-octadece-nameid

References:

1. Jao C, Lin W, Wu Y, et al. Isolation, structure elucidation, and synthesis of cytotoxic tryptanthrin analogues from Phaius mishmensis[J]. Journal of natural products, 2008, 71(7): p. 1275–1279.
2. Barnes J P, Putnam A R, Burke B A, et al. Isolation and characterization of allelochemicals in rye herbage[J]. Phytochemistry, 1987, 26(5):1385-1390.
3. D'Abrosca B, Dellagreca M, Fiorentino A, et al. Structure elucidation and phytotoxicity of C_{13} nor-isoprenoids from Cestrum parqui[J]. Phytochemistry, 2004, 65(4): 497-505.
4. Abdolali A, Jvad M. Unexpected approach to the synthesis of 2-phenyl quinoxalines and pyrido[2,3-b]pyrazines via a regioselective reaction[J]. Helvetica Chimica Acta, 2013, 96(1): 124-129.
5. Koálová D, Hrochová V, Suchý V, et al. Two pyrrole acids from Berberis koreana[J]. Phytochemistry, 1992, 31(10): 3669-3670.
6. Katade SR, Pawar PV, Tungikar VB, et al. Larvicidal activity of bis (2-ethylhexyl) benzene-1,2-dicarboxylate from Sterculia guttata seeds against two mosquito species[J]. Chemistry & Biodiversity, 2006, 3(1): 49-53.
7. Zhang W, Lou H, Li G, et al. A new triterpenoid from Entodon okamurae broth[J]. Journal of Asian Natural Products Research, 2003, 5(3): 189-195.
8. Wu P, Hsu Y, Jao C. Indole alkaloids from Cephalanceropsis gracilis[J]. Journal of Natural Products, 2006, 69(10): 1467-1470.
9. DellaGreca M, Di Marino C, Zarrelli A, D'Abrosca B. Isolation and phytotoxicity of apocarotenoids from Chenopodium album[J]. Journal of natural products, 2004, 67(9):1492-1495.
10. Gutierrez-Lugo M, Woldemichael GM, Singh MP, et al. Isolation of three new naturally occurring compounds from the culture of Micromonospora sp. P1068[J]. Natural Product Research, 2005, 19(7): 645-652.
11. Duan H, Takaishi Y, Momota H, et al. Immunosuppressive constituents from Saussurea medusa[J]. Phytochemistry, 2002, 59(1): 85-90.
12. Jones L, Bartholomew B, Latif Z, et al. Constituents of Cassia laevigata[J]. Fitoterapia, 2000, 71(5): 580-583.
13. Liu YH, Qing GW, et al. Studies on chemical constituents in root of Isatis indigotica[J]. Chinese Traditional and Herbal Drugs, 2002, 33(2): 97-99.
14. Yang Q, Ye G. A new c-glucoside from Commelina communis[J], Chemistry of Natural Compounds, 2009, 45(1): 59-60.
15. Marques MR, Stüker C, Kichik N, et al. Flavonoids with prolyl oligopeptidase inhibitory activity isolated from Scutellaria racemosa Pers[J]. Fitoterapia, 2010, 81(6): 552-556.

16. Francisco AM, David M, Alberto O, et al. Isolation and synthesis of allelochemicals from gramineae: Benzoxazinones and related compounds[J]. Journal of Agricultural and Food Chemistry, 2006, 54(4): 991-1000.
17. Xie H, Liang Y, Xue J. Secondary metabolites of the phytopathogen Peronophythora litchii[J]. Natural Product Communications, 2010, 5(2): 245-248.
18. Yang H, Hou A, Mei S, et al. Constituents of Clerodendrum bungei[J]. Journal of Asian Natural Products Research, 2002, 4(3): 165-169.
19. Hacıbekiroğlu, I. and Kolak, U. Antioxidant and anticholinesterase constituents from the petroleum ether and chloroform extracts of Iris suaveolens[J]. Phytotherapy Ressearch, 2011, 25(4): 522-529.
20. Chen Y, Fan CL, Wang Y, et al. Chemical constituents from flowers of Abelmoschus manihot[J]. Zhongguo Zhong Yao Za Zhi. 2018, 43(10): 2091-2096
21. Yang SY, Li ZL, et al. Chemical constituents from roots of Isatis indigotica[J]. Chinese Traditional and Herbal Drugs. 2017, 48(14): 2827-2842
22. Liu Y, Ou YF, et al. Chemical constituents in the leaves of Baphicacanthus cusia (Nees) Bremek[J]. Chinese Journal of Medicinal Chemistry. 2009, 19(4): 273-275
23. Xie P, Zhang Y, Wang X, et al. Antithrombotic effect and mechanism of Rubus spp. Blackberry[J]. Food & function, 2017, 8(5): 2000-2012.
