# Supplementary material for: Combining the In Silico and In Vitro Assays to Identify Strobilanthes cusia Kuntze Bioactives against Penicillin-Resistant Streptococcus pneumoniae
Source: Pharmaceuticals (Basel). 2023 Jan 10;16(1):105. doi: 10.3390/ph16010105 (PMC9863409; doi:10.3390/ph16010105)
Supplement: Supplementary file 1 [file pharmaceuticals-16-00105-s001.zip › Table S8. A scheme of Preparation of Isolated Compounds from S. cusia leaves.pptx]

## Slide 1
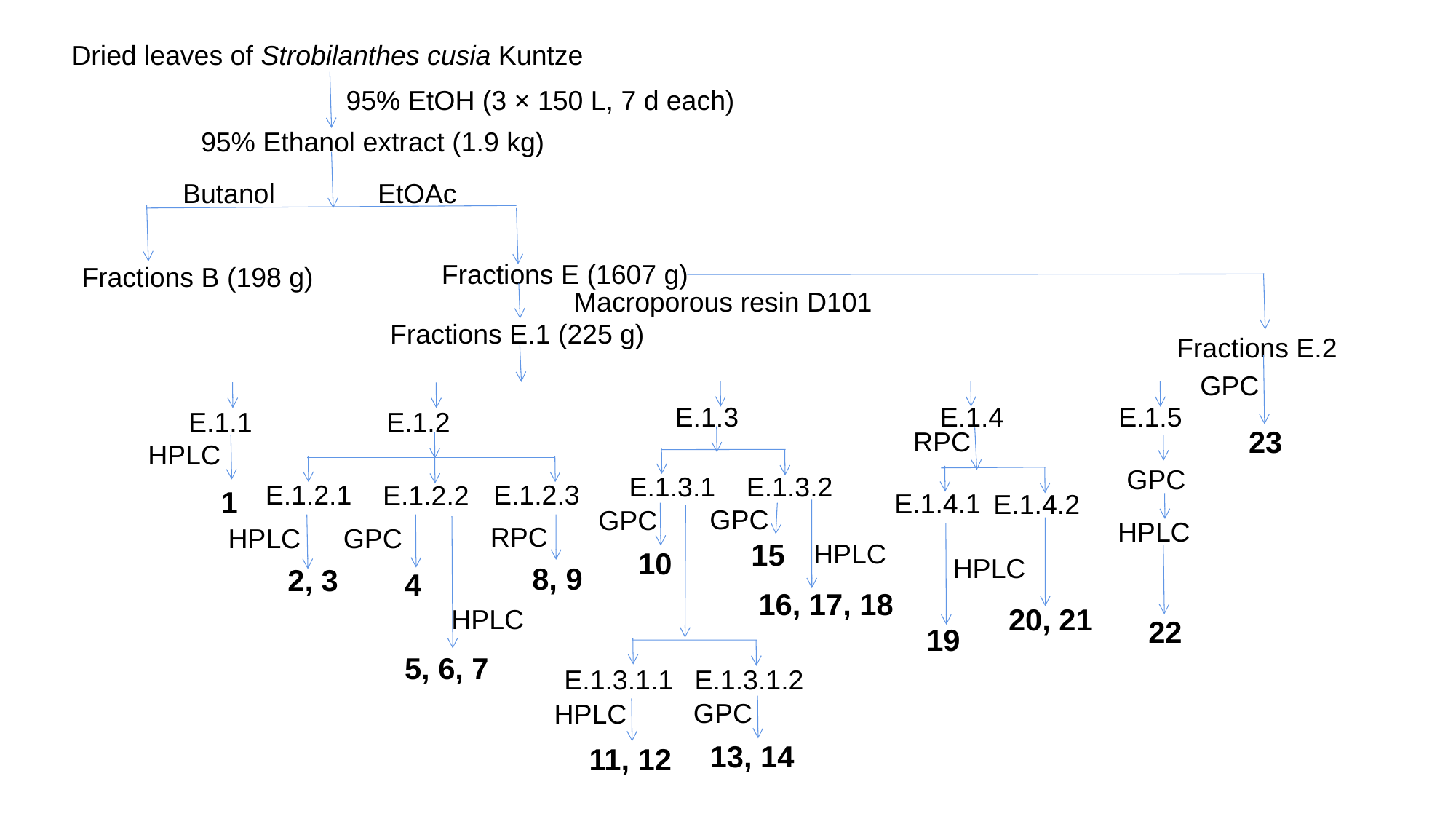

Dried leaves of Strobilanthes cusia Kuntze
95% EtOH (3 × 150 L, 7 d each)
95% Ethanol extract (1.9 kg)
Butanol
EtOAc
Fractions E (1607 g)
Fractions B (198 g)
Macroporous resin D101
Fractions E.1 (225 g)
Fractions E.2
GPC
E.1.3
E.1.4
E.1.5
 E.1.1
E.1.2
23
RPC
HPLC
GPC
E.1.3.1
E.1.3.2
E.1.2.1
E.1.2.3
E.1.2.2
1
E.1.4.1
E.1.4.2
GPC
GPC
HPLC
RPC
HPLC
GPC
15
HPLC
10
HPLC
8, 9
2, 3
4
16, 17, 18
20, 21
HPLC
22
19
5, 6, 7
E.1.3.1.1
E.1.3.1.2
GPC
HPLC
13, 14
11, 12
